# Behavioral Phenotyping of Arts Engagement Using 20 Years of the American Time Use Survey

**DOI:** 10.1111/nyas.70284

**Published:** 2026-04-23

**Authors:** Jessica K. Bone, Feifei Bu, Jill K. Sonke, Daisy Fancourt

**Affiliations:** ^1^ Research Department of Behavioural Science and Health, Institute of Epidemiology & Health Care University College London London UK; ^2^ Center for Arts in Medicine University of Florida Gainesville Florida USA

**Keywords:** arts, culture, leisure, trends

## Abstract

Arts and cultural engagement have been linked to health and wellbeing but there has not been any national monitoring of arts and cultural behaviors in the United States. Given its potential benefits, understanding how engagement is changing is vital. We used the American Time Use Survey (ATUS), a continuous cross‐sectional survey (2003−2023) measuring activities during the last 24 h. We estimated rates of arts engagement on an average day; determined how long people spent on the arts; explored with whom activities were done; identified where activities were done; examined potential disparities across different subgroups of the population; and described the time trends in these measures. In this nationally representative sample (*n* = 236,270), daily arts engagement rates were stable from 2003 to 2023, with small underlying increases in participatory engagement (dancing/performing/arts/crafts/writing) and slight declines in receptive engagement (attending performing arts/museums/watching dancing). There were less consistent trends in time spent on the arts. People increasingly did arts activities alone at home. There were disparities in engagement, with differing trends according to sex, age, and income. To understand population‐level engagement, arts behaviors should be monitored nationally. Tracking different activities separately is crucial, alongside the factors influencing participation.

## Graphical abstract

This study uses data from the American Time Use Survey to explore changes in daily arts engagement in the United States from 2003 to 2023. Over this period, there were small increases in participatory arts engagement and slight declines in receptive arts engagement on the average day. Daily rates of listening to or making music remained stable over time.

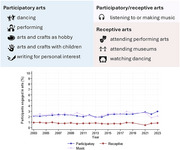

## 1 Introduction

The arts are complex human behaviors, comprising different types of creative practices, multiple modes of engagement, and diverse art forms [1]. We use the term arts engagement to encompass the full range of everyday artistic, creative, and cultural behaviors. This includes a wide range of heterogeneous activities and can involve active participation (e.g., painting, making music, storytelling, dancing), receptive consumption of art that has been created and is now experienced by an audience (e.g., going to a museum, cultural event, theater, or concert), and broader creative activities (e.g., gardening, cooking, rituals, hobby groups). These activities are ubiquitous in human culture. In the last few decades, there has been increasing empirical research on arts and health, with emerging evidence that the arts could be used to manage and treat illness, prevent ill health, and in wider health promotion [2, 3]. Epidemiological research has linked arts engagement with a range of positive outcomes, such as lower risk of developing depression [4, 5, 6], chronic pain [7], and cognitive decline [8, 9, 10], better self‐assessed general health [11, 12], and enhanced psychological [13, 14] and social wellbeing [3, 15, 16]. While epidemiological research relies on observational data, and studies have been limited by inconsistent findings and heterogeneous definitions of the arts [17, 18, 19, 20], there is increasing application of causal inference methods in this field [21]. Triangulation of findings using different statistical methods has led to proposals that engaging with the arts could be considered a health‐enhancing behavior [22, 23, 24, 25].

In addition to potential links with health, arts activities provide opportunities for creative expression, develop cultural understanding, and can bring communities together [2, 3]. It is thus important to understand levels of engagement in the population. Detailed phenotyping is particularly critical to identify disparities or high‐risk populations that face barriers to arts engagement. National and international surveillance systems, including the World Health Organization (WHO) Global Health Observatory [26], allow monitoring of behaviors such as physical activity [27], substance use [28], and diet [29]. This monitoring has been important for setting recommendations and targets. However, similar targets for arts engagement do not yet exist. We have relatively little understanding of arts behaviors—who engages, in which art forms, where, and with whom. Arts engagement is not included in international surveillance systems, making monitoring challenging. Additionally, the lack of data on long‐term trends makes it difficult to assess how behaviors might be changing over time, the impact of changes to funding and federal policies, and what kinds of barriers and enablers people face to participation. Monitoring patterns of arts engagement is thus critical to understand the implications of previous policy, support future policy, and identify populations who may not be accessing the arts.

We have previously explored trends in arts engagement from 1993 to 2016 in the United States, finding fluctuations in rates of attending art performances and decreases in creative group membership over time [30]. However, our study was limited to the narrow definition of arts engagement used in the General Social Survey (GSS), and the types of arts activities measured changed over time, which may have influenced findings. Similar issues affect the Survey of Public Participation in the Arts (SPPA; National Endowment for the Arts) and public opinion polls by Americans for the Arts (AFTA), which provide snapshots of arts behaviors in the United States every 3–5 years [31, 32]. The SPPA has historically focused on a limited number of more exclusive arts activities (e.g., classical music, opera, ballet), and the SPPA, GSS, and AFTA all ask people about engagement in the arts over the last year [30, 31, 32, 33, 34], which is subject to large recall biases.

The limitations of existing measures of arts engagement become most apparent when compared to the measurement of health behaviors. Physical activity is generally measured in minutes, with different types of activity separated. Diet is measured using portion sizes for various food groups or 24‐h dietary recalls. Asking whether someone has been to a museum in the past year, in contrast, lacks detail. Other behavioral aspects of engagement, including when in the day, where, and with whom activities are done, are rarely measured. Existing evidence thus does not have the granularity needed to understand everyday arts engagement. Additionally, more comparisons are needed of data from recent years to before the COVID‐19 pandemic, which hugely disrupted the provision of, funding for, and engagement with the arts [35, 36].

In this study, we aimed to address these limitations by analyzing time use survey data, which identifies, quantifies, and classifies people's behaviors within a specific 24‐h window, providing more detail than previous studies, substantially reducing recall bias, and enabling analysis of patterns in behaviors over multiple years [37]. Specifically, we analyzed data from the American Time Use Survey (ATUS), which includes over 10,000 individuals each year, providing nationally representative estimates for an average day in the United States. ATUS is particularly valuable for understanding arts behaviors, as it captures an accurate, detailed, and reliable picture of what people did, where they were, and who they were with minute to minute over the last day. We aimed to: (1) estimate rates of engagement in the arts on an average day; (2) determine how long people spent on the arts, both overall and for different art forms; (3) explore who arts activities were done with; (4) identify where arts activities were done; (5) examine potential disparities by exploring variation across different subgroups of the population; and (6) describe the national trends in these measures from 2003 to 2023.

## 2 Materials and Methods

### 2.1 Sample

ATUS is a continuous cross‐sectional survey, covering all residents of private households in the United States aged 15 and over [37]. Individuals are randomly selected from a subset of households that have completed their eighth month of interviews for the Current Population Survey (CPS). One individual per household is invited to ATUS 2 months after completing this CPS interview.

Data collection began in 2003, with data currently available through 2023. We excluded 2020 due to complications during the COVID‐19 pandemic (data collection was paused for part of the year, so weights for 2020 cannot be combined with other years). Approximately 26,400 people were eligible per year, but response rates declined over time (57.8%−35.8%) [37]. Each participant was interviewed once. ATUS excluded participants with incomplete responses. This left a total of 236,357 participants. Following the UCL Centre for Time Use Research's recommendations, we excluded participants who did not report: spending time on sleep/rest/personal care/eating/drinking (*n* = 15); at least one change in location (*n* = 59); and at least one change in the presence of others (*n* = 13). This left a final analytical sample of 236,270 participants.

All ATUS participants gave informed consent. This analysis has Institutional Review Board approval from the University of Florida (IRB202401080) and ethical approval from University College London Research Ethics Committee (project 18839/001).

### 2.2 Measures

ATUS asked participants to recall their activities over 24 h, beginning at 4 am on the day prior to the interview and ending at 4 am on the day of the interview. Participants were randomly assigned a day of the week on which to complete the survey, with 10% of the sample allocated to each weekday and 25% to each weekend day. Weights then account for this nonuniform distribution and differing response rates across days of the week, so that measures can be estimated for an average day. Information on secondary activities (activities that are done at the same time as the primary activity) was not collected, except for childcare. Participants reported every activity they took part in during the 24‐h period, including where they were and whom they were with. Activities were coded using a standard lexicon, verified by two coders, and classified within a three‐tiered system, from broad to detailed categories, including examples.

#### 2.2.1 Arts and Cultural Engagement

ATUS classified specific activities into overarching categories, from which we identified 11 types of art activities: (1) dancing; (2) watching dancing; (3) performing; (4) extracurricular music and performance activities; (5) arts and crafts as a hobby; (6) writing for personal interest; (7) listening to or making music (not the radio); (8) attending performing arts; (9) attending museums; (10) arts and crafts with household children; and (11) arts and crafts with household nonhousehold children. A broad range of specific activities were included within each activity type, as outlined in Table S1.

**FIGURE 1:**
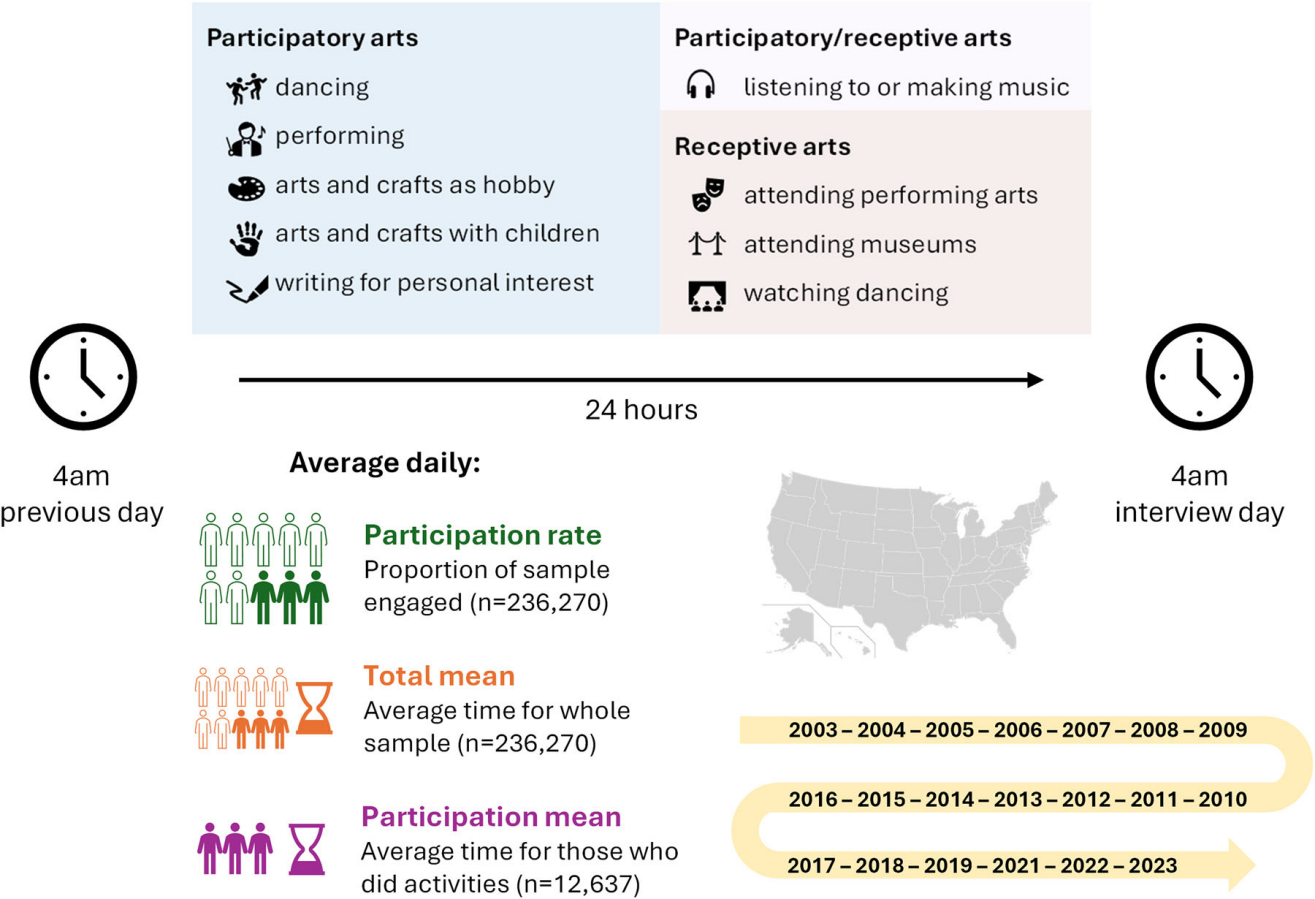
Dataset characterization and measures of arts engagement.

The UNESCO framework for measuring arts and cultural participation recommends including a broad range of activities that encompass diverse cultural practices [38]. Applying this framework to the ATUS data, we created three sets of indicators: (1) engagement in any arts activities; (2) engagement in participatory versus receptive arts domains; and (3) engagement in each of the specific arts activities listed above (Figure 1). The participatory art domain included active creation of art, specifically through dancing, performing, extracurricular music and performance activities, arts and crafts (as a hobby and with children), and writing for personal interest. The receptive art domain included consuming art that has been created, namely, watching dancing, attending performing arts, and attending museums. Listening to or making music was examined separately as ATUS included examples of both participatory and receptive activities within this category (e.g., singing, listening to recorded music).

Reading for personal interest has been explored separately because it was more common than other arts activities and showed very different time trends [39], indicating that it is a distinct behavior that follows different daily patterns and should not be combined with receptive arts activities. Screen‐based activities (attending movies/film and watching television/movies) were not included in the main analyses because they were also far more common than other arts activities, and there is mixed evidence on whether these activities are associated with positive or negative health outcomes [15, 40, 41]. Other related but excluded activities were arts and entertainment not elsewhere classified (which included nonarts activities), income‐generating activities and travel related to arts and entertainment (as not strictly leisure), and serving at volunteer events and cultural activities (where the main activity was serving rather than the arts).

For each type of arts engagement, we measured whether participants engaged in the activity (i.e., spent ≥1 min on it) and the total time (minutes) spent on the activity during the diary day. It is important to note that these indices measure arts engagement on the average day. This daily engagement rate thus reflects the intensity of everyday involvement in the arts and is likely much lower than weekly, monthly, or annual prevalence rates. While it is assumed that daily patterns correspond to the prevalence over longer periods, measuring engagement on the average day may structurally underestimate engagement, particularly for infrequent behaviors (e.g., receptive arts). We, therefore, do not aim to estimate the longer‐term prevalence of engagement at the population level.

We also categorized with whom arts were done into six social contexts: alone, with partner or spouse, with child or grandchild, with other family member, with a friend/colleague/neighbor/acquaintance, and with multiple people. Finally, we categorized where arts were done into seven locations: own home, other's home, workplace, library, school, other community location (e.g., outdoors, place of worship, while traveling, other), or multiple locations. Arts and cultural venues (e.g., theater, music venue, museum, historic site) were not included in the list of potential locations, so these were likely recorded in the “other” category.

#### 2.2.2 Individual Characteristics

Demographic, socioeconomic, and health‐related information was collected either during the ATUS interview or from earlier CPS interviews. We included sex (male, female), age group (15−24 years, 25–65 years, 66 years and over), race (White, Black, Asian, Other [including American Indian, Alaskan Native, Hawaiian/Pacific Islander, multiple racial groups]), education (high school or less, college, undergraduate, postgraduate), annual family income (quartiles: less than $30,000, $30,000−$59,999, $60,000−$99,999, $150,000 and over), metropolitan status (non‐metropolitan area, metropolitan area), and disability status (no disability, disability that prevents work). Sex, age, metropolitan, and disability status were measured in the ATUS interview. Race, education, and income were measured in the CPS interview, 2–5 months before the ATUS interview (mean = 3.04, standard deviation [SD] = 0.57). Further sociodemographic characteristics are included in Table 1.

**TABLE 1:** Characteristics of the sample.

Characteristic	Proportion
Sex	
Male	48%
Female	52%
Age	
15−24 years	17%
25−65 years	67%
66 years and over	16%
Race	
White	81%
Black	12%
Asian	4%
Other	2%
Marital status	
Married	53%
Widowed/divorced/separated	17%
Never married	30%
Child under 18 in household	39%
Education	
High school or less	45%
College	25%
Undergraduate	19%
Postgraduate	11%
Employment status	
Employed	63%
Unemployed	5%
Not in labor force	16%
Retired	16%
Annual family income	
Less than $30,000	25%
$30,000−$59,999	28%
$60,000−$99,999	24%
$150,000 and over	23%
Metropolitan status	
Non‐metropolitan area	16%
Metropolitan area	84%
Disability that prevents work	4%
	**Mean (SD)**
Household size	2.98 (1.56)
Number of children in household	0.75 (1.13)

*Note: N* = 236,270. Results are weighted and based on 20 imputed datasets.

### 2.3 Statistical Analysis

We aimed to test whether arts engagement has changed from 2003 to 2023. First, we explored the proportion of participants who did arts activities (participation rate), the average time spent on arts overall (total mean), and the average time spent on arts just for participants who undertook these activities (participation mean) [42]. We used a series of regression models to examine time trends. Poisson regression with robust standard errors estimated prevalence ratios, testing whether participation rates changed over time, for arts overall and each domain and activity separately. Linear regression tested whether the amount of time spent on arts activities (total mean, participation mean) changed over time, overall, and for each domain and activity.

Next, we explored social context and activity location. Given the low frequency of arts activities done with different people, we tested whether the proportion of participants who did activities alone (vs. with others) changed over time using Poisson regression with robust standard errors. Similarly, as arts activities were mostly done at home, we tested whether the proportion of participants who did activities only in the home (own home/others’ homes vs. outside home) differed over time using Poisson regression with robust standard errors. Finally, we examined whether time trends differed across population groups. We added interactions between participation year and individual characteristics for the overall participation rate and participation mean in separate models. We then repeated analyses stratified by individual characteristics.

In all models, time (year) was treated as a linear exposure. We ran two additional sets of models for each outcome to test this assumption: (1) including a quadratic effect of time, and (2) treating time as categorical (the most complex model possible). We compared models using fit statistics (Akaike information criterion, Bayesian information criterion), Wald tests (quadratic vs. linear model), and likelihood‐ratio tests (categorical vs. continuous model). Wald tests indicated that including quadratic terms did not improve model fit (*p*>0.05), except for activity location. Although likelihood‐ratio tests indicated that treating time as categorical did improve model fit over including it as a continuous linear exposure for most outcomes, likelihood‐ratio tests may not be valid for models with probability weights or robust standard errors, as assumptions are violated. Given this, and to avoid overfitting and aid interpretation, time was treated as linear in all models.

ATUS generated weights to account for complex sampling, day of the week, and response rates across demographics and days. We used these weights to generate estimates for an average day representative of the US civilian noninstitutionalized population aged 15 and over. Missingness was generally low (<6%; Table S2), although ATUS replaced missing income data using values from previous CPS waves for 9% of participants. To account for data that were still missing, we used multiple imputation by chained equations [43]. We generated 20 imputed data sets using ordered logistic and logistic regression according to variable type. The imputation model included all variables used in analyses, sampling weights, and auxiliary variables (Table S2). Separate imputation models were used for overall arts indices and individual activities due to collinearity. Findings from imputed analyses did not differ from complete case analyses (Tables S8−S13), so imputed results are reported. All analyses were performed using Stata 18 [44].

#### 2.3.1 Sensitivity Analyses

In sensitivity analyses, we first tested whether outliers in the number of minutes spent on art activities (participation mean) influenced findings. Top‐coding outliers at three standard deviations above the mean (416 min) or at the 99th percentile (513 min) did not alter the results, so analyses are reported without outliers removed or recoded.

Second, we assessed whether including screen‐based activities in the receptive art domain altered the findings. Screen‐based activities were (a) attending movies/film and (b) watching television/movies (not religious or religious). In the main complete case analyses, we report results for receptive engagement, including screen‐based activities, as well as the results separately for these activities. This included descriptive statistics and the Poisson and linear regression models, testing time trends.

## 3 Results

We included 236,270 individuals aged 15 and over (mean age = 45.14, SD = 18.63) who completed ATUS once between 2003 and 2023 (excluding 2020). After weighting, 52% were female, 81% were of White race, 53% were married, and 63% were employed (Table 1).

### 3.1 Overall Engagement

**FIGURE 2:**
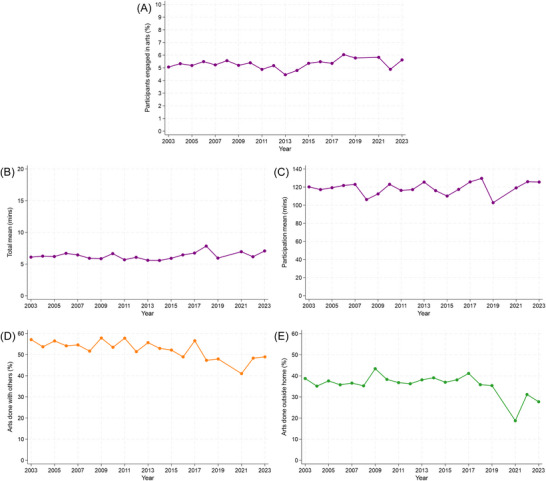
Overall engagement in arts activities from 2003 to 2023 (excluding 2020 due to the COVID‐19 pandemic). (A) Participation rate: proportion of the sample who engaged in the arts. (B) Total mean: average time spent on the arts in the full sample. (C) Participation mean: average time spent on arts just for participants who undertook these activities. (D) Proportion of participants who did arts activities with others. (E) Proportion of participants who did arts activities outside the home.

We focused on engagement in arts activities on an average day. Overall, between 2003 and 2023, engagement equated to 6 min per day (SD = 37) spent on any arts activities (Table S3). However, only 5% of participants engaged in one or more arts activities; the remaining 95% reported no daily engagement. The 12,637 participants who did arts activities spent an average of 1 h and 59 min (SD = 109; median = 90) on these activities per day. Figure 2 (panel A−C) shows how these measures fluctuated between 2003 and 2023. The overall averages were very similar to those just for 2023 (Supplementary Materials). Regression models showed no associations between year (treated as linear) and these measures, indicating that there was no linear change in daily participation from 2003 to 2023 (Tables S4 and S5).

### 3.2 Social Context

Most participants did arts activities alone (50%), with multiple people (15%), or with a friend (14%). It was less common to do arts with a partner (8%), other family member (7%), or child (6%). Figure 2D shows how participation with others changed over time. In 2003, 57% of participants doing arts activities did them with others. This dropped to a low of 41% in 2021 and recovered to 49% by 2023. Using Poisson regression with robust standard errors, the proportion who did arts with others (vs. alone) decreased by 1% per year from 2003 to 2023 (prevalence ratio [PR] = 0.99, 95% confidence interval [CI] = 0.98, 0.99, *p*<0.001).

### 3.3 Location

Most participants did arts activities in their own home (60%) or other community locations (26%; e.g., outdoors, place of worship, while traveling). Doing arts in other places was less common: 4% were in others’ homes, 4% at school, 2% in the workplace, 0.1% at a library, and 4% in multiple locations. In 2003, 39% of those who did arts activities engaged outside the home, but this decreased to 19% in 2021, before then recovering to 28% by 2023 (Figure 2E). The proportion of participants who reported doing arts activities outside the home (vs. at home) decreased over time (PR = 0.99, 95% CI = 0.98, 0.99, *p*<0.001).

### 3.4 Specific Activities

Examining domains separately, 2% of participants did participatory activities (dancing, performing, arts and crafts, writing), 1% receptive (attending performing arts, museums, watching dancing), and 2% listened to or made music on an average day. Among those who engaged, 2 h and 8 min (SD = 116) was spent on participatory activities per day, 2 h and 41 min (SD = 100) on receptive, and 1 h and 26 min (SD = 88) listening to/making music, although there was substantial variability between participants.

**FIGURE 3:**
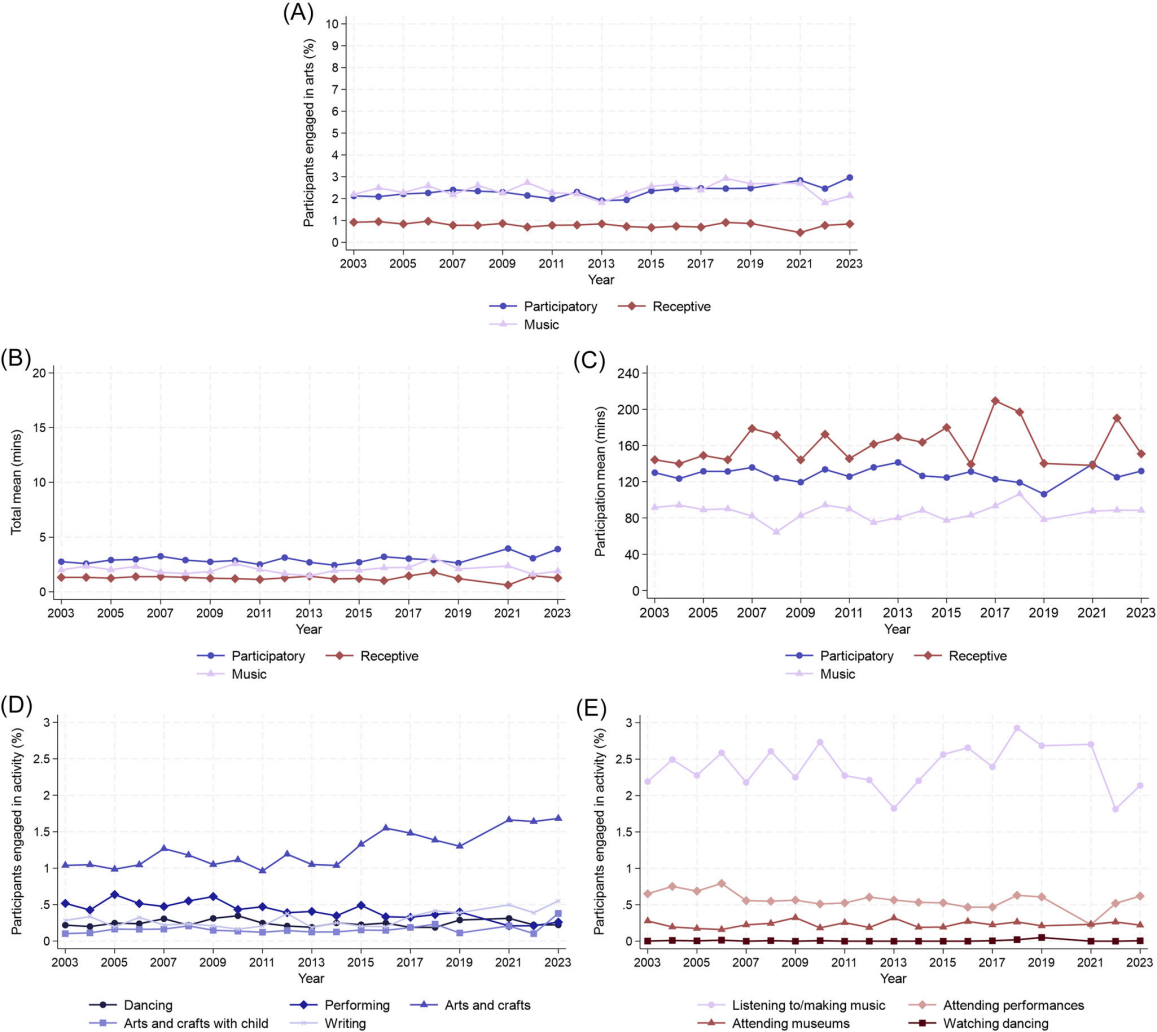
Overall engagement by domain and activity participation rates from 2003 to 2023 (excluding 2020). (A) Participation rates (proportion of sample who engaged) for participatory activities, receptive activities, and listening to/making music. (B) Total means (average time spent in the full sample) for participatory activities, receptive activities, and listening to/making music. (C) Participation means (average time spent just for those who engaged) for participatory activities, receptive activities, and listening to/making music. (D) Participation rates for specific participatory activities. (E) Participation rates for specific receptive activities.

Poisson regression models showed that from 2003 to 2023, daily participatory engagement increased slightly, while daily receptive engagement decreased (Figure 3A). Rates of listening to/making music did not change over time. Linear regression showed that trends in time spent on domains also differed, with evidence for increases in the time spent engaging among the whole sample just for participatory activities (Figure 3B), and increases in the time spent engaging only among those who engaged for receptive activities (Figure 3C).

Daily participation rates for specific activities varied but were generally low (0.01% watched dancing; 2% listened to/made music; Figure 3, panel D,E). Yet, participation rates increased by 4% per year for writing (PR = 1.04, 95% CI = 1.02, 1.06, *p*<0.001) and 3% for arts and crafts both as a hobby (PR = 1.03, 95% CI = 1.02, 1.04, *p*<0.001) and with children (PR = 1.03, 95% CI = 1.00, 1.05, *p* = 0.026). In contrast, daily engagement decreased by 4% per year for performing (PR = 0.96, 95% CI = 0.95, 0.98, *p*<0.001) and 2% for attending performing arts (PR = 0.98, 95% CI = 0.97, 0.99, *p* = 0.004). The time spent on each activity per day was also low (Figure S1), ranging from 55 min (arts and crafts with children) to 2 h and 44 min (attending performing arts). In linear regression models, only time spent attending performing arts was associated with year, with evidence for an additional 1.61 min spent attending performances each year (coef = 1.61, 95% CI = 0.30, 2.92, *p* = 0.016).

### 3.5 The Role of Individual Characteristics

**FIGURE 4:**
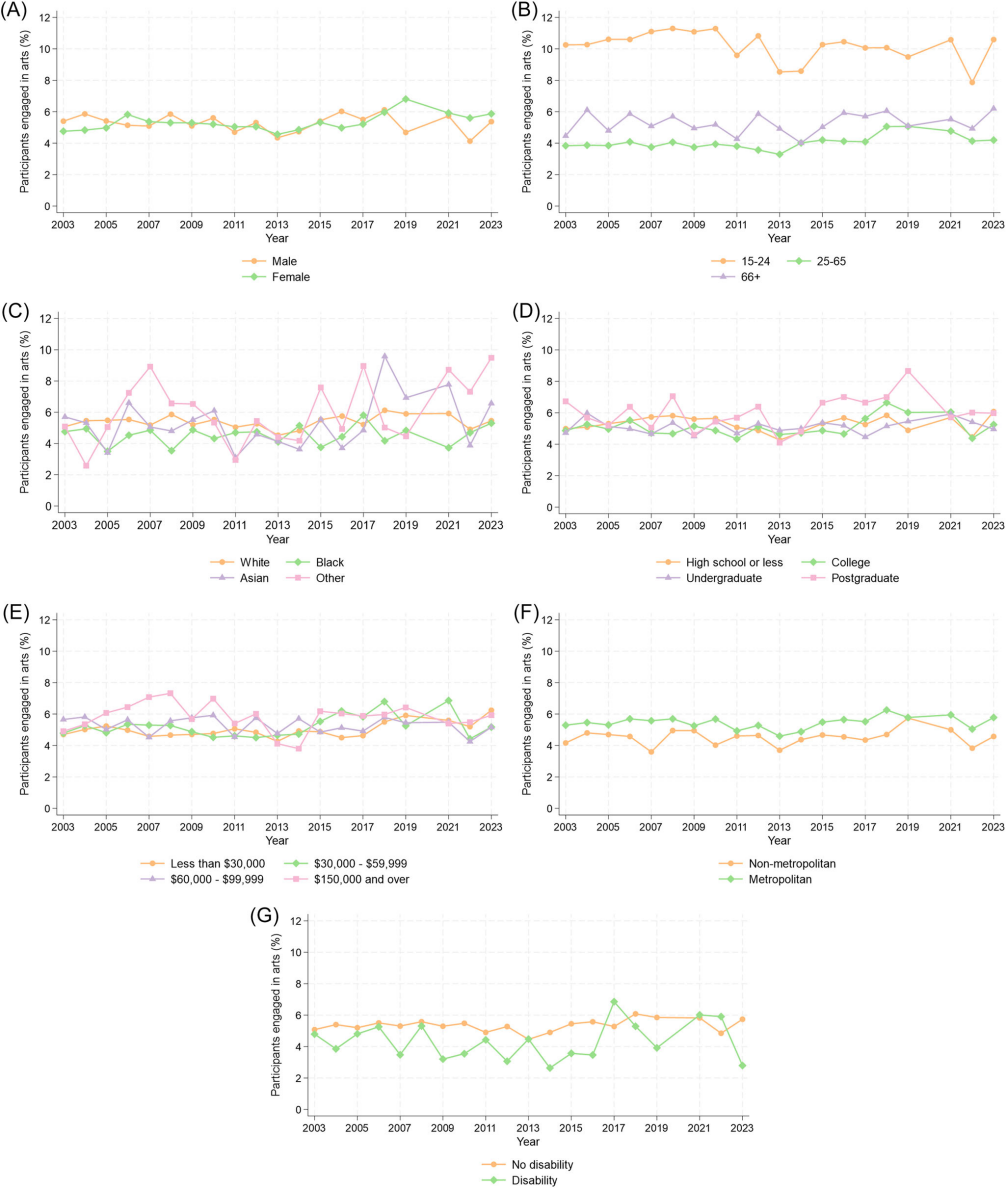
Differential trends in participation rates from 2003 to 2023 (excluding 2020) stratified by individual characteristics. (A) Sex: male, female. (B) Age group: 15–24 years, 25–65 years, 66 years and over. (C) Race: White, Black, Asian, Other (including American Indian, Alaskan Native, Hawaiian/Pacific Islander, mixed race). (D) Education: high school or less, college, undergraduate, postgraduate. (E) Annual family income quartiles: less than $30,000, $30,000−$59,999, $60,000−$99,999, $150,000 and over. (F) Metropolitan status: non‐metropolitan area, metropolitan area. (G) Disability status: no disability, disability that prevents work.

We then explored the role of individual characteristics in overall daily engagement. Looking at participation rates descriptively (Figure 4), engagement was highest in the youngest (aged 15–24) individuals, those living in metropolitan areas, and those with no disability. In contrast, participation rates were similar across males and females, racial groups, and people with different levels of education and income. The time spent on art activities was less clearly differentiated across population groups (Figure S2), with some indication that it was higher in males, the oldest individuals, those living in non‐metropolitan areas, and those with a disability.

We explored whether time trends differed across population groups, adding interactions between year (treated as linear) and individual characteristics to regression models used in the main analysis (Table S6). We then repeated analyses stratified by the individual characteristics (Table S7). Time trends in daily participation rates differed according to sex, age group, and income (Figure 4). Exploring sex differences, daily participation increased in females over time but did not change in males. Females’ average participation rate started at 4.8% in 2003 and peaked at 6.8% in 2019. For age groups, participation rates were highest in those aged 15–24 and remained stable both in this group and those aged 66 and over, but increased in those aged 25–65 years (3.8% in 2003, peaked at 5.1% in 2018). Looking at income, there was weak evidence for increases in people with an annual family income under $60,000, but this was not present in those with more income. For example, those earning $30,000−$59,999 had a daily participation rate of 4.8% in 2003, which peaked at 6.9% in 2021.

In contrast, trends in time spent on the arts by those who engaged only differed across racial groups. The amount of time spent on arts activities on an average day was stable in participants of White, Black, and Asian races, but decreased from 2003 to 2023 in those of American Indian, Alaskan Native, Hawaiian/Pacific Islander, or multiple racial groups (Figure S2).

### 3.6 Sensitivity Analyses

Screen‐based activities (attending movies/film and watching television/movies) were more common than other receptive activities; the participation rate for all receptive activities increased to 79% after these were included (Table S9). This was mainly driven by watching television/movies (participation rate = 79%) compared to attending movies/film (1%). Among those who engaged, 3 h and 7 min (SD = 136) was spent on receptive and screen‐based activities on an average day. Poisson regression showed that from 2003 to 2023, daily receptive and screen‐based engagement decreased very slightly (Table S10). However, as in the main analyses, linear regression showed increases in the time spent engaging among those who engaged in receptive and screen‐based activities (Table S11).

## 4 Discussion

This study provides the first behavioral phenotyping of daily arts engagement in the United States from 2003 to 2023. Overall rates of engagement, when summarized across a wide range of arts and creative activities, were stable over time. This appeared to be a result of a slight increase in daily participatory engagement (dancing, performing, arts and crafts, writing) combined with a small decrease in daily receptive engagement (attending performing arts, museums, watching dancing). Despite this decrease, the amount of time spent on receptive activities by those who participated on an average day did increase slightly over the 20‐year period. There were also increases in the time spent on participatory activities among the whole sample, but this was likely driven by rising participatory engagement rates. Most people did arts activities alone and at home, and the proportion doing so increased over time. There was some evidence for disparities in engagement, with differing trends in daily engagement according to sex, age, and family income. Sex differences increased over time, with daily participation rates stable in males but increasing in females, whereas disparities due to age and income may have decreased over time.

The lack of change in daily engagement overall was surprising given previous evidence for declines in creative group membership [30], attending visual and performing arts [31], and reading [39] during this period, alongside the general perception that arts participation is declining in the United States [45]. This could be because we focused on engagement in the arts on an average day, as opposed to monthly or annual prevalence rates measured in previous studies. While daily engagement is likely to correspond to longer‐term prevalence, daily measures are more likely to capture the most engaged individuals within the population and may show differing time trends to longer‐term prevalence. Our findings could also be a result of including a wide range of arts and creative activities, including activities such as making a TikTok video or podcast (categorized by ATUS as “arts and crafts as a hobby”), allowing our definition of arts engagement to reflect newer ways of engaging in the arts that have not been captured in previous studies. Yet, despite emerging evidence that the arts could have benefits for health and wellbeing [22, 23, 24, 25], alongside efforts to increase access to the arts [46, 47], daily engagement levels have not changed. In contrast, average daily participation rates in sports and exercise in the United States rose by 4% from 2003 to 2015 [48], and use of tobacco products declined 33% among young adults and 22% among adults from 2002 to 2022 [49]. Daily arts engagement thus does not appear to be following the same pattern as established health behaviors.

Underlying the overall stability, there were small changes in specific activities, including declines in performing and attending performing arts. The latter is consistent with SPPA data suggesting falling attendance of visual and performing arts in the United States from 2017 to 2022 [31]. A surprisingly low proportion of arts activities were done with others or outside the home, and this also declined from 2003 to 2023. These declines may reflect reductions in arts funding in the United States over the last 20 years [50], with fewer resources available to support community‐based arts venues and run performing arts events. The COVID‐19 pandemic is another important factor. By far the lowest rates of receptive engagement were in 2021, following pandemic lockdowns. There were also substantial dips in activities done with others or outside the home in 2021. Daily engagement had somewhat recovered by 2022, but it did not reach prepandemic levels, potentially reflecting consequences of the COVID‐19 pandemic, including the closure of many arts venues, additional barriers such as having to book in advance, and changes in behavioral norms. In ATUS, examples of performing arts did not explicitly include digital engagement (e.g., watching a recording of a play), meaning some engagement may have been missed. It was previously shown that the majority of those engaging in the arts in 2022 did so through electronic or digital media [31]. The apparent decline in daily engagement may thus be a result of shifts to more digital engagement, which could also explain the decline in activities done with others and outside the home. However, in sensitivity analyses, we still found evidence for a small decline in receptive engagement after including screen‐based activities, indicating that digital engagement is following similar patterns to other receptive activities.

Our findings suggest that males, working‐age adults, those living in non‐metropolitan areas, and those with a disability have the lowest daily participation rates. These groups may experience the most systemic barriers to regular arts participation. Although our findings on sex, urbanicity, and disability status are consistent with previous evidence, we did not find the expected large disparities according to education or income [30, 51, 52, 53, 54]. In fact, there was some evidence for increases in daily engagement just for people with an annual family income under $60,000. Those earning $30,000−$59,999 had a daily participation rate of 4.8% in 2003, which then peaked at 6.9% in 2021. Despite being small absolute differences, these changes are meaningful when considered in relation to the low engagement rates, representing a 44% relative increase. Additionally, despite working‐age adults having the lowest participation rates, daily engagement did increase in this group over time. These reductions in income‐ and age‐related disparities could indicate that strategies to improve access to the arts may have had some success, helping people to overcome barriers such as cost, difficulty of getting to a venue, and lack of time [55, 56]. Despite this, our findings indicate that increasing sex differences and sustained disparities according to residential area and disability status require further attention. Research should explore the underlying drivers of these inequities, particularly as they may result from unjust systems, practices, or norms.

In addition to highlighting specific individual‐level disparities to address, our findings demonstrate the need for initiatives to increase regular arts engagement at the population level, particularly targeting receptive activities that are done with others outside the home. Activities done outside the home and with others may provide opportunities for social interactions, developing social identities and social support, physical activity, and exposure to nature, all of which could lead to improvements in health and wellbeing [57]. Yet, an individual's arts engagement is determined by many interconnected factors [58]. Changing engagement is complex and requires simultaneous intervention at multiple levels. Monitoring is vital to understand the impact of interventions to increase engagement. Fortunately, the recently established Arts Indicators Project, led by the National Arts Statistics and Evidence‐based Reporting Center (NASERC), began doing so in 2024 [59]. The project aims to provide regular statistics on the arts in the United States, using data from robust, publicly accessible, and nationally representative sources (e.g., Annual Business Survey, American Community Survey, ATUS, Current Population Survey, SPPA). In addition, five arts behavior questions were added to the US Census Bureau's Household Pulse Survey in spring 2024 [60]. Analyses are planned to explore trends over time, the role of individual characteristics, and regional variations in arts participation, cultural assets, and art education, which will further extend our findings and be valuable for those working in arts policy [59].

This study has several strengths. We included over 236,000 people, with weights making estimates nationally representative of an average day in the United States. Although response rates declined over time, ATUS measured all daily activities, so there may be less selection bias than surveys explicitly focused on the arts. Time‐use surveys are also less susceptible to recall bias than other surveys that require reporting over longer periods. ATUS allowed us to use a broader definition of arts than previous surveys. While overarching categories remained consistent over time, specific examples of activities within these domains were updated, which is important to keep up with emerging ways of participating in the arts. In contrast, previous research has not been able to compare changes in participatory engagement over time due to changes in measures [31]. We also provide the first population‐level data on trends in social context and location of everyday arts engagement.

However, there are some limitations of the ATUS activity classifications. Participation in many art forms is infrequent when measured daily, so ATUS had to group specific activities into larger categories, such as “attending performing arts” or “arts and crafts as a hobby,” to gain reliable estimates. This prevented a more detailed investigation of some engagement types, including the separation of listening to and making music into its receptive and participatory elements. ATUS also included some specific activities that, while describing broader creative or cultural activities, have not traditionally been defined as “arts” (e.g., auctioneering, visiting the zoo, organizing a coin collection with a child). However, in this study, we have used a broad definition of the arts, with activity categories that encompass diverse cultural practices (as recommended by UNESCO) [38]. These activities are, therefore, consistent with our definition. We excluded some activity categories as they included nonarts activities (e.g., arts and entertainment not elsewhere classified), but this could mean we missed some forms of engagement. We also could not separate some digital/virtual consumption of arts from other activities, which may mean we further underestimated overall rates of engagement, as this is the most popular mode of arts participation [31]. We chose to focus on overall engagement because, despite the large sample size, many types of engagement were still rare. This also meant we could not explore the social context or location of activities separately. Further detailed phenotyping of specific activities is needed when more daily data become available. Engagement on the average day cannot be compared with monthly or annual prevalence rates. Additionally, our measures of sex (male, female; due to availability in ATUS) and race (White, Black, Asian, Other; due to small numbers in non‐White groups) were overly simplistic. This approach conflates experiences across diverse sex, racial, and ethnic groups, which might be problematic as these groups may not have equal access to the arts [30]. Future research should collect more nuanced data on sex and further explore the role of race and ethnicity in arts participation.

## 5 Conclusion

Overall, we found small increases in average daily rates of participatory arts engagement and small declines in daily receptive arts engagement over the last 20 years in the United States. People have become less likely to do arts activities with others or outside the home on the average day. Ultimately, to understand engagement at the population level, arts behaviors should be integrated into national and international surveillance systems. The NASERC Arts Indicators Project will be key for monitoring arts behaviors in the United States. Our findings indicate that tracking different arts activities separately is crucial, and it will be equally important to measure where activities are done and who with, as well as the demographic and socioeconomic characteristics influencing participation.

## Author Contributions

J.K.B., D.F., and F.B. conceptualized the study and developed the analytical plan. J.K.B. performed analyses, with input from D.F. and F.B., and drafted the manuscript. All coauthors contributed to the interpretation and reporting of findings and read and approved the final version of the manuscript.

## Conflicts of Interest

No authors report any conflicts of interest.

## Supporting information




**Supporting Materials**: nyas70284‐sup‐0001‐SuppMat.docx

## Data Availability

This study used publicly available ATUS data, available from: https://www.bls.gov/tus/data.htm. Statistical code is available on OSF: https://doi.org/10.17605/OSF.IO/8NBXD.
