# Supplementary material for: Behavioral Phenotyping of Arts Engagement Using 20 Years of the American Time Use Survey
Source: Ann N Y Acad Sci. 2026 Apr 23;1558:e70284. doi: 10.1111/nyas.70284 (PMC13104588; doi:10.1111/nyas.70284)
Supplement: Supplementary file 1 — Supporting Materials: nyas70284‐sup‐0001‐SuppMat.docx [file NYAS-1558-0-s001.docx]

**Supplementary Materials**

| **Content** | **Page** |
| --- | --- |
| Table S1. ATUS arts and cultural activities | 1 |
| Table S2. Missing data in analytical sample | 2 |
| Main analysis results | 2 |
| Table S3. Average engagement levels | 2 |
| Figure S1. Proportion of time spent on specific activities | 2 |
| Table S4. Poisson regressions for participation rates | 3 |
| Table S5. Linear regressions for time spent on activities | 3 |
| Table S6. Interactions with individual characteristics | 4 |
| Table S7. Stratified by individual characteristics | 4 |
| Figure S2. Stratified participation means | 5 |
| Complete case results | 6 |
| Table S8. Sample characteristics | 6 |
| Table S9. Average engagement levels | 7 |
| Table S10. Poisson regressions for participation rates | 7 |
| Table S11. Linear regressions for time spent on activities | 8 |
| Table S12. Interactions with individual characteristics | 8 |
| Table S13. Stratified by individual characteristics | 9 |
| Engagement in 2023 | 9 |

# Table S1. ATUS activity codes for arts and cultural activities, with third-tier (detailed) activities shown within the first- and second-tier categories and the examples given to participants for each tier 3 activity as of 2023.

| **Tier 1** | **Tier 2** | **Tier 3** | **Examples provided (from 2023)** |
| --- | --- | --- | --- |
| Sports exercise and recreation | Participating in sports, exercise, and recreation | Dancing | - |
|  | Attending sports/recreational events | Watching dancing | - |
| Volunteer activities | Participating in performance and cultural activities | Performing | acting in a performance (volunteer), dancing in a performance (volunteer), performing music (volunteer), emceeing a charity function (volunteer), auctioneering a benefit auction (volunteer), practicing for a performance (volunteer) |
| Education | Extracurricular school activities (except sports) | Extracurricular music and performance activities | Attending band practice, attending choir practice as extracurricular school activity, attending play practice |
| Socialising relaxing and leisure | Relaxing and leisure | Arts and crafts as a hobby | Scrapbooking/making a scrapbook, making holiday/other decorations, videotaping/photography/model making/jewelry making, making pottery/sculpting/wood working, taking pictures/snapshots/photographs, making a TikTok video, making Halloween costumes (for self), dyeing Easter eggs, artistic painting, making Christmas decorations, sketching/drawing, making podcast |
|  |  | Writing for personal interest | writing in diary/journal (personal interest), writing lyrics, blogging (personal interest), writing stories (personal interest), editing (personal interest) |
|  |  | Listening to / playing music (not radio) | Listening to recorded music/records/CDs/DVDs/tapes, playing musical instrument (leisure), singing/singing karaoke/Christmas caroling, listening to someone play the piano, composing music, tuning musical instruments |
|  | Arts and entertainment (other than sports) | Attending performing arts | attending comedy club, attending opera, attending a concert, attending the ballet, attending dance troupe performances, attending plays, attending musicals, attending the theatre, attending jazz bar |
|  |  | Attending museums | attending an art gallery, visiting the zoo, attending exhibitions, attending an arboretum, attending a botanical garden |
| Caring for and helping household members | Caring for and helping household children | Arts and crafts with household children | making holiday decorations with household child, helping household child make arts and crafts, organizing coin collection with household child, building model planes with household child |
| Caring for and helping non-household members | Caring for and helping non-household children | Arts and crafts with non-household children | making holiday decorations w/non-household child, helping non-household child make arts and crafts, organizing coin collection with non-household child, building model planes with non-household child |

# Table S2. Missing data in the full analytical sample.

| **Variable** | **Missing** | **Proportion** |
| --- | --- | --- |
| Activity engagement | - | - |
| Year | - | - |
| Sex | - | - |
| Age | - | - |
| Race | - | - |
| Education | - | - |
| Family income | 13,693 | 5.80% |
| Metropolitan status | 1792 | 0.76% |
| Disability status | 61 | 0.03% |

*Note.* N=236,270. Auxiliary variables: Hispanic status, cohabitation, marital status, household size, presence and number of children under 18 in the household, labor force status, disability status, provision of care or assistance for older adults, time between the ATUS and CPS completion, diary day, US State, US region.

**Main analysis results**

# Table S3. Rates of arts engagement, averaged over time.

| **Activity** | **Participation rate** | **Total mean (SD)** | **Participation mean (SD)*** |
| --- | --- | --- | --- |
| Any arts | 5% | 6.31 (36.67) | 118.79 (109.33) |
| Any participatory | 2% | 2.97 (26.17) | 127.82 (116.19) |
| Any receptive | 1% | 1.28 (16.84) | 161.39 (100.00) |
| Dancing | 0.2% | 0.31 (7.84) | 125.92 (97.27) |
| Performing | 0.4% | 0.50 (9.43) | 120.50 (84.07) |
| Arts and crafts | 1% | 1.77 (21.33) | 140.61 (128.73) |
| Arts and crafts with child | 0.2% | 0.09 (2.80) | 55.49 (41.72) |
| Writing | 0.3% | 0.31 (8.50) | 101.14 (117.26) |
| Listening to/making music | 2% | 2.06 (18.99) | 86.39 (88.43) |
| Attending performances | 0.6% | 0.92 (14.29) | 163.61 (97.93) |
| Attending museum | 0.2% | 0.35 (8.61) | 149.53 (98.91) |
| Watching dancing | 0.01% | 0.01 (1.69) | 148.53 (153.17) |

*Note.* N=236,270. Percentages, means, and standard deviations weighted and based on 20 imputed datasets. *Participation mean calculated only for those who participated in each activity.

# **Figure S1.** Proportion of total engagement spent on specific arts activities from 2003 to 2023 (excluding 2020).

# Table S4. Poisson regression models testing the associations between survey year and arts participation rates.

| **Outcome** | **N** | **Prevalence ratio** | **95% CI** | **p value** |
| --- | --- | --- | --- | --- |
| **Overall engagement** |  |  |  |  |
| Participation rate | 236,270 | 1.00 | 1.00, 1.01 | 0.099 |
| Arts done with others | 12,635 | **0.99** | **0.99, 0.99** | **<0.001** |
| Arts done outside home | 12,637 | **0.99** | **0.98, 0.99** | **<0.001** |
| **Domain participation rates** |  |  |  |  |
| Participatory | 236,270 | **1.01** | **1.01, 1.02** | **<0.001** |
| Receptive | 236,270 | **0.99** | **0.98, 1.00** | **0.032** |
| Listening to/making music | 236,270 | 1.00 | 0.99, 1.01 | 0.909 |
| **Individual activity participation rates** | | | | |
| Dancing | 236,270 | 1.00 | 0.98, 1.02 | 0.866 |
| Performing | 236,270 | **0.96** | **0.95, 0.98** | **<0.001** |
| Arts and crafts | 236,270 | **1.03** | **1.02, 1.04** | **<0.001** |
| Arts and crafts with child | 236,270 | **1.03** | **1.00, 1.05** | **0.026** |
| Writing | 236,270 | **1.04** | **1.02, 1.06** | **<0.001** |
| Listening to/making music | 236,270 | 1.00 | 0.99, 1.01 | 0.909 |
| Attending performances | 236,270 | **0.98** | **0.97, 0.99** | **0.004** |
| Attending museum | 236,270 | 1.00 | 0.99, 1.02 | 0.565 |
| Watching dancing | 236,270 | 1.06 | 0.97, 1.15 | 0.211 |

# Table S5. Linear regression models testing the associations between survey year and time spent on arts and cultural activities.

| **Outcome** | **N** | **Coefficient** | **95% CI** | **P value** |
| --- | --- | --- | --- | --- |
| **Overall engagement** | | | | |
| Arts total mean | 236,270 | 0.03 | 0.00, 0.07 | 0.084 |
| Arts participation mean | 12,637 | 0.19 | -0.27, 0.65 | 0.418 |
| **Domain total means** |  |  |  |  |
| Participatory | 236,270 | **0.03** | **0.01, 0.06** | **0.014** |
| Receptive | 236,270 | 0.00 | -0.02, 0.01 | 0.538 |
| Listening to/making music | 236,270 | 0.00 | -0.02, 0.02 | 0.699 |
| **Domain participation means** |  |  |  |  |
| Participatory | 5629 | -0.18 | -0.88, 0.53 | 0.624 |
| Receptive | 2391 | **1.10** | **0.07, 2.13** | **0.036** |
| Listening to/making music | 5088 | 0.13 | -0.48, 0.73 | 0.677 |
| **Individual activity participation means** | | | | |
| Dancing | 616 | -1.70 | -3.55, 0.14 | 0.070 |
| Performing | 831 | 0.78 | -0.57, 2.13 | 0.258 |
| Arts and crafts | 2908 | -0.56 | -1.66, 0.53 | 0.312 |
| Arts and crafts with child | 491 | 0.01 | -0.74, 0.77 | 0.969 |
| Writing | 705 | 0.16 | -1.36, 1.68 | 0.834 |
| Listening to/making music | 5088 | 0.13 | -0.48, 0.73 | 0.677 |
| Attending performances | 1649 | **1.61** | **0.30, 2.92** | **0.016** |
| Attending museum | 745 | 0.36 | -1.22, 1.93 | 0.656 |
| Watching dancing | 19 | -3.52 | -15.89, 8.85 | 0.577 |

# Table S6. Tests of whether the associations between survey year and overall arts engagement differed according to survey year (interaction terms from regression models).

|  | **Participation rate** (n=236,270) | | | **Participation mean** (n=12,637) | | |
| --- | --- | --- | --- | --- | --- | --- |
|  | Prevalence  ratio | 95% CI | p value | Coef | 95% CI | p value |
| Sex | **1.01** | **1.00, 1.02** | **0.006** | 0.35 | -0.58, 1.27 | 0.461 |
| Age: 25 – 65 years | **1.02** | **1.01, 1.03** | **0.002** | -0.85 | -2.04, 0.34 | 0.161 |
| Age: 66 years and over | 1.01 | 1.00, 1.02 | 0.070 | -0.29 | -1.72, 1.15 | 0.694 |
| Race: Black | 1.00 | 0.99, 1.02 | 0.860 | -0.13 | -1.47, 1.22 | 0.851 |
| Race: Asian | 1.01 | 0.99, 1.04 | 0.213 | 1.55 | -0.90, 4.00 | 0.215 |
| Race: Other | 1.02 | 0.99, 1.05 | 0.194 | **-4.24** | **-7.24, -1.23** | **0.006** |
| Education: College | 1.01 | 1.00, 1.02 | 0.278 | -0.42 | -1.65, 0.81 | 0.500 |
| Education: Undergraduate | 1.00 | 0.99, 1.01 | 0.674 | -0.45 | -1.58, 0.68 | 0.438 |
| Education: Postgraduate | 1.01 | 1.00, 1.02 | 0.209 | -0.58 | -1.90, 0.75 | 0.395 |
| Income: $30,000 - $59,999 | 1.00 | 0.99, 1.01 | 0.865 | 0.21 | -1.19, 1.61 | 0.771 |
| Income: $60,000 - $99,999 | **0.99** | **0.98, 1.00** | **0.051** | -0.83 | -2.12, 0.46 | 0.210 |
| Income: $150,000 and over | 0.99 | 0.98, 1.00 | 0.066 | 0.46 | -0.90, 1.81 | 0.507 |
| Metropolitan status | 1.00 | 0.99, 1.01 | 0.918 | 0.37 | -0.88, 1.61 | 0.562 |
| Disability status | 1.00 | 0.98, 1.02 | 0.804 | 1.35 | -1.68, 4.37 | 0.383 |

# Table S7. Tests of whether the associations between survey year and overall arts engagement differed according to survey year (stratified regression models).

|  | | **Participation rate** (n=236,270) | | | **Participation mean** (n=12,637) | | |
| --- | --- | --- | --- | --- | --- | --- | --- |
|  | | PR | 95% CI | p value | Coef | 95% CI | p value |
| Sex | Male | 1.00 | 0.99, 1.00 | 0.408 | 0.05 | -0.69, 0.80 | 0.889 |
|  | Female | **1.01** | **1.00, 1.01** | **0.001** | 0.40 | -0.15, 0.95 | 0.153 |
| Age | 15 – 24 years | 0.99 | 0.99, 1.00 | 0.175 | 0.57 | -0.48, 1.63 | 0.284 |
|  | 25 – 65 years | **1.01** | **1.01, 1.02** | **<0.001** | -0.27 | -0.83, 0.28 | 0.329 |
|  | 66 years and over | 1.00 | 1.00, 1.01 | 0.231 | 0.29 | -0.69, 1.26 | 0.564 |
| Race | White | 1.00 | 1.00, 1.01 | 0.329 | 0.30 | -0.20, 0.80 | 0.236 |
|  | Black | 1.00 | 0.99, 1.02 | 0.622 | 0.17 | -1.08, 1.42 | 0.789 |
|  | Asian | 1.02 | 0.99, 1.04 | 0.141 | 1.85 | -0.55, 4.25 | 0.131 |
|  | Other | 1.02 | 0.99, 1.05 | 0.145 | **-3.94** | **-6.90, -0.97** | **0.009** |
| Education | High school or less | 1.00 | 0.99, 1.01 | 0.939 | 0.45 | -0.34, 1.24 | 0.266 |
|  | College | 1.01 | 1.00, 1.01 | 0.137 | 0.02 | -0.92, 0.97 | 0.960 |
|  | Undergraduate | 1.00 | 0.99, 1.01 | 0.540 | 0.00 | -0.81, 0.81 | 1.000 |
|  | Postgraduate | 1.01 | 1.00, 1.02 | 0.098 | -0.13 | -1.20, 0.94 | 0.813 |
| Annual family income | Less than $30,000 | 1.01 | 1.00, 1.02 | 0.097 | 0.31 | -0.65, 1.26 | 0.528 |
|  | $30,000 - $59,999 | **1.01** | **1.00, 1.02** | **0.047** | 0.52 | -0.49, 1.52 | 0.313 |
|  | $60,000 - $99,999 | 1.00 | 0.99, 1.00 | 0.280 | -0.52 | -1.37, 0.33 | 0.232 |
|  | $150,000 and over | 1.00 | 0.99, 1.00 | 0.321 | 0.77 | -0.19, 1.72 | 0.116 |
| Metropolitan status | Non-metropolitan area | 1.00 | 0.99, 1.01 | 0.657 | -0.10 | -1.25, 1.04 | 0.859 |
|  | Metropolitan area | 1.00 | 1.00, 1.01 | 0.202 | 0.27 | -0.23, 0.76 | 0.296 |
| Disability status | No disability | 1.00 | 1.00, 1.01 | 0.117 | 0.14 | -0.32, 0.61 | 0.541 |
|  | Disability | 1.01 | 0.99, 1.03 | 0.562 | 1.49 | -1.50, 4.48 | 0.328 |

*Note.* PR: Prevalence ratio from Poisson regression model. Coef: Coefficient from linear regression model.

# Figure S2. Differential trends in participation means (time spent on activities by those who participated) from 2003 to 2023 (excluding 2020) stratified by individual characteristics. A) Sex: male, female. B) Age group: 15-24 years, 25-65 years, 66 years and over. C) Race: White, Black, Asian, Other (including American Indian, Alaskan Native, Hawaiian/Pacific Islander, mixed race). D) Education: high school or less, college, undergraduate, postgraduate. E) Annual family income quartiles: less than $30,000, $30,000 - $59,999, $60,000 - $99,999, $150,000 and over. F) Metropolitan status: non-metropolitan area, metropolitan area. G) Disability status: no disability, disability that prevents work.

**Complete case results**

# Table S8. Characteristics of the complete case sample (weighted).

| **Characteristic** | **Proportion** |
| --- | --- |
| Sex |  |
| Male | 48% |
| Female | 52% |
| Age |  |
| 15-24 years | 17% |
| 25-65 years | 67% |
| 66 years and over | 16% |
| Race |  |
| White | 81% |
| Black | 12% |
| Asian | 4% |
| Other | 2% |
| Marital status |  |
| Married | 52% |
| Widowed/divorced/separated | 17% |
| Never married | 31% |
| Child under 18 in household | 40% |
| Education |  |
| High school or less | 45% |
| College | 25% |
| Undergraduate | 19% |
| Postgraduate | 11% |
| Employment status |  |
| Employed | 63% |
| Unemployed | 5% |
| Not in labour force | 17% |
| Retired | 15% |
| Annual family income |  |
| Less than $30,000 | 25% |
| $30,000 - $59,999 | 28% |
| $60,000 - $99,999 | 24% |
| $150,000 and over | 23% |
| Metropolitan status |  |
| Non-metropolitan area | 16% |
| Metropolitan area | 84% |
| Disability prevents work | 4% |
|  | **Mean (SD)** |
| Household size | 2.98 (1.56) |
| Number of children in household | 0.75 (1.13) |

*Note.* N=220,777.

# Table S9. Rates of arts engagement, averaged over time, in complete cases only.

| **Activity** | **Participation rate** | **Total mean (SD)** | **Participation mean (SD)*** |
| --- | --- | --- | --- |
| Any arts | 11,831 (5%) | 6.35 (36.83) | 119.09 (109.65) |
| Any participatory | 4,760 (2%) | 2.99 (26.27) | 127.40 (116.45) |
| Any receptive | 1,759 (1%) | 1.30 (17.02) | 162.66 (100.62) |
| Any receptive (inc. TV/film) | 177,825 (79%) | 166.82 (171.58) | 187.47 (136.40) |
| Dancing | 581 (0.2%) | 0.30 (7.76) | 124.52 (96.34) |
| Performing | 933 (0.4%) | 0.49 (9.38) | 120.32 (83.58) |
| Arts and crafts | 2,802 (1%) | 1.79 (21.48) | 141.05 (129.30) |
| Arts and crafts with child | 466 (0.2%) | 0.09 (2.81) | 55.40 (41.21) |
| Writing | 669 (0.3%) | 0.31 (8.57) | 101.74 (117.39) |
| Listening to/making music | 4,760 (2%) | 2.06 (18.97) | 86.29 (88.27) |
| Attending performances | 1,543 (0.6%) | 0.94 (14.45) | 164.77 (98.60) |
| Attending museum | 702 (0.2%) | 0.35 (8.68) | 150.92 (99.24) |
| Watching dancing | 17 (0.01%) | 0.01 (1.71) | 148.59 (163.60) |
| Attending movies/film | 2,929 (1%) | 1.53 (15.17) | 135.23 (65.92) |
| Watching TV/movies | 176,195 (79%) | 163.99 (171.17) | 167.82 (129.35) |

*Note.* N=220,777. Percentages, means, and standard deviations weighted. *Participation mean calculated only for those who participated in each activity (n shown under participation rate).

# Table S10. Poisson regression models testing the associations between survey year and arts participation rates, in complete cases only.

| **Outcome** | **N** | **Prevalence ratio** | **95% CI** | **p value** |
| --- | --- | --- | --- | --- |
| **Overall engagement** |  |  |  |  |
| Participation rate | 220,777 | 1.00 | 1.00, 1.01 | 0.078 |
| Arts done with others | 11,831 | 0.99 | 0.99, 0.99 | <0.001 |
| Arts done outside home | 11,831 | 0.99 | 0.98, 0.99 | <0.001 |
| **Domain participation rates** |  |  |  |  |
| Participatory | 220,777 | 1.01 | 1.01, 1.02 | <0.001 |
| Receptive | 220,777 | 0.99 | 0.98, 1.00 | 0.032 |
| Receptive (inc. TV/film) | 220,777 | 0.997 | 0.997, 0.998 | <0.001 |
| Listening to/making music | 220,777 | 1.00 | 0.99, 1.01 | 0.762 |
| **Individual activity participation rates** | | | | |
| Dancing | 220,777 | 1.00 | 0.98, 1.02 | 0.855 |
| Performing | 220,777 | 0.96 | 0.94, 0.97 | <0.001 |
| Arts and crafts | 220,777 | 1.03 | 1.02, 1.04 | <0.001 |
| Arts and crafts with child | 220,777 | 1.03 | 1.00, 1.05 | 0.042 |
| Writing | 220,777 | 1.04 | 1.02, 1.06 | <0.001 |
| Listening to/making music | 220,777 | 1.00 | 0.99, 1.01 | 0.762 |
| Attending performances | 220,777 | 0.98 | 0.97, 0.99 | 0.003 |
| Attending museum | 220,777 | 1.01 | 0.99, 1.02 | 0.438 |
| Watching dancing | 220,777 | 1.07 | 0.98, 1.17 | 0.122 |
| Attending movies/film | 220,777 | 0.95 | 0.94, 0.95 | <0.001 |
| Watching TV/movies | 220,777 | 0.997 | 0.997, 0.998 | <0.001 |

# Table S11. Linear regression models testing the associations between survey year and time spent on arts and cultural activities for those who participated in these activities, in complete cases only.

| **Outcome** | **N** | **Coefficient** | **95% CI** | **P value** |
| --- | --- | --- | --- | --- |
| **Overall engagement** | | | | |
| Arts total mean | 220,777 | 0.03 | 0.00, 0.07 | 0.080 |
| Arts participation mean | 11,831 | 0.17 | -0.30, 0.65 | 0.471 |
| **Domain participation means** | | | | |
| Participatory | 5,272 | -0.22 | -0.96, 0.52 | 0.567 |
| Receptive | 2,242 | 0.96 | -0.10, 2.03 | 0.076 |
| Receptive (inc. TV/film) | 177,825 | 1.30 | 1.12, 1.47 | <0.001 |
| Listening to/making music | 4,760 | 0.23 | -0.37, 0.84 | 0.454 |
| **Individual activity participation means** | | | | |
| Dancing | 581 | -1.50 | -3.40, 0.39 | 0.119 |
| Performing | 933 | 0.74 | -0.67, 2.14 | 0.304 |
| Arts and crafts | 2,722 | -0.66 | -1.83, 0.51 | 0.272 |
| Arts and crafts with child | 466 | 0.04 | -0.73, 0.81 | 0.919 |
| Writing | 669 | -0.22 | -1.79, 1.35 | 0.781 |
| Listening to/making music | 4,760 | 0.23 | -0.37, 0.84 | 0.454 |
| Attending performances | 1,543 | 1.54 | 0.19, 2.90 | 0.025 |
| Attending museum | 702 | 0.10 | -1.55, 1.75 | 0.904 |
| Watching dancing | 17 | -4.42 | -20.35, 11.51 | 0.586 |
| Attending movies/film | 2,929 | 0.99 | 0.62, 1.36 | <0.001 |
| Watching TV/movies | 176,195 | 1.36 | 1.19, 1.54 | <0.001 |

# Table S12. Tests of whether the associations between survey year and overall arts engagement differed according to survey year (interaction terms from regression models), in complete cases only.

|  | **Participation rate** (n=220,777) | | | **Participation mean** (n=11,831) | | |
| --- | --- | --- | --- | --- | --- | --- |
|  | Prevalence ratio | 95% CI | p value | Coefficient | 95% CI | p value |
| Sex | 1.01 | 1.00, 1.02 | 0.008 | 0.29 | -0.67, 1.25 | 0.556 |
| Age: 25 – 65 years | 1.02 | 1.00, 1.03 | 0.004 | -0.96 | -2.17, 0.26 | 0.122 |
| Age: 66 years and over | 1.01 | 1.00, 1.02 | 0.072 | -0.66 | -2.18, 0.85 | 0.389 |
| Race: Black | 1.00 | 0.99, 1.02 | 0.937 | -0.23 | -1.66, 1.20 | 0.756 |
| Race: Asian | 1.02 | 0.99, 1.04 | 0.166 | 1.40 | -1.06, 3.87 | 0.264 |
| Race: Other | 1.03 | 0.99, 1.06 | 0.112 | -4.04 | -7.31, -0.78 | 0.015 |
| Education: College | 1.01 | 0.99, 1.02 | 0.330 | -0.23 | -1.49, 1.04 | 0.727 |
| Education: Undergraduate | 1.00 | 0.99, 1.01 | 0.666 | -0.57 | -1.76, 0.61 | 0.341 |
| Education: Postgraduate | 1.01 | 0.99, 1.02 | 0.291 | -0.58 | -1.96, 0.80 | 0.411 |
| Income: $30,000 - $59,999 | 1.00 | 0.99, 1.01 | 0.916 | 0.16 | -1.24, 1.56 | 0.820 |
| Income: $60,000 - $99,999 | 0.99 | 0.98, 1.00 | 0.034 | -0.90 | -2.19, 0.40 | 0.174 |
| Income: $150,000 and over | 0.99 | 0.97, 1.00 | 0.026 | 0.49 | -0.89, 1.86 | 0.487 |
| Metropolitan status | 1.00 | 0.99, 1.01 | 0.691 | 0.43 | -0.87, 1.74 | 0.515 |
| Disability status | 1.00 | 0.98, 1.02 | 0.792 | 1.19 | -1.92, 4.30 | 0.454 |

# Table S13. Tests of whether the associations between survey year and overall arts engagement differed according to survey year (stratified regression models), in complete cases only.

|  | | **Participation rate** (n=220,777) | | | **Participation mean** (n=11,831) | | |
| --- | --- | --- | --- | --- | --- | --- | --- |
|  | | PR | 95% CI | p value | Coef | 95% CI | p value |
| Sex | Male | 1.00 | 0.99, 1.00 | 0.500 | 0.07 | -0.70, 0.84 | 0.861 |
|  | Female | 1.01 | 1.00, 1.02 | 0.001 | 0.36 | -0.21, 0.93 | 0.221 |
| Age | 15 – 24 years | 0.99 | 0.99, 1.00 | 0.253 | 0.65 | -0.41, 1.72 | 0.229 |
|  | 25 – 65 years | 1.01 | 1.00, 1.02 | <0.001 | -0.30 | -0.88, 0.28 | 0.306 |
|  | 66 years and over | 1.01 | 1.00, 1.01 | 0.155 | -0.01 | -1.08, 1.06 | 0.985 |
| Race | White | 1.00 | 1.00, 1.01 | 0.282 | 0.30 | -0.21, 0.81 | 0.255 |
|  | Black | 1.00 | 0.99, 1.02 | 0.676 | 0.07 | -1.26, 1.41 | 0.917 |
|  | Asian | 1.02 | 1.00, 1.04 | 0.101 | 1.70 | -0.71, 4.11 | 0.167 |
|  | Other | 1.03 | 1.00, 1.06 | 0.078 | -3.75 | -6.98, -0.51 | 0.023 |
| Education | High school or less | 1.00 | 0.99, 1.01 | 0.822 | 0.41 | -0.40, 1.23 | 0.319 |
|  | College | 1.01 | 1.00, 1.01 | 0.142 | 0.19 | -0.78, 1.16 | 0.701 |
|  | Undergraduate | 1.00 | 0.99, 1.01 | 0.451 | -0.16 | -1.02, 0.70 | 0.714 |
|  | Postgraduate | 1.01 | 1.00, 1.02 | 0.129 | -0.16 | -1.28, 0.95 | 0.772 |
| Annual family income | Less than $30,000 | 1.01 | 1.00, 1.02 | 0.052 | 0.31 | -0.65, 1.28 | 0.524 |
|  | $30,000 - $59,999 | 1.01 | 1.00, 1.02 | 0.027 | 0.48 | -0.53, 1.49 | 0.356 |
|  | $60,000 - $99,999 | 1.00 | 0.99, 1.00 | 0.302 | -0.58 | -1.45, 0.28 | 0.184 |
|  | $150,000 and over | 0.99 | 0.99, 1.00 | 0.221 | 0.80 | -0.17, 1.78 | 0.107 |
| Metropolitan status | Non-metropolitan area | 1.01 | 0.99, 1.02 | 0.339 | -0.18 | -1.39, 1.02 | 0.767 |
|  | Metropolitan area | 1.00 | 1.00, 1.01 | 0.227 | 0.25 | -0.26, 0.77 | 0.335 |
| Disability status | No disability | 1.00 | 1.00, 1.01 | 0.078 | 0.14 | -0.34, 0.62 | 0.568 |
|  | Disability | 1.00 | 0.98, 1.02 | 0.923 | 1.33 | -1.75, 4.40 | 0.397 |

*Note.* PR: Prevalence ratio from Poisson regression model. Coef: Coefficient from linear regression model.

# Engagement in 2023

From our analytical sample, 8543 participants completed the ATUS survey in 2023. Among these participants, the average time spent on any arts activities in the last day was 7 minutes. Overall, 6% engaged in any arts activities, with those who participated spending an average of 119 minutes on the arts. Of the 474 participants who engaged in the arts, 28% did so outside the home, and 49% did so with others.

Looking at art domains separately, 3% did participatory activities, 1% receptive, and 2% listened to or made music. Among those who engaged in each of these domains, 2 hours and 12 minutes were spent on participatory activities, 2 hours and 31 minutes on receptive, and 1 hour and 28 minutes on listening to/making music.

In 2023, engagement was highest in the youngest individuals (aged 15-24) and lowest in working age adults (25-65). Additionally, participants with a disability were less likely to have engaged than those without a disability. However, there was no evidence for differences in overall engagement according to sex, race, education, family income, or metropolitan status. There was no consistent evidence for differences in time spent on the arts by those who participated according to individual characteristics, likely due to small group sizes (only 474 participants engaged in the arts).
